# Maternity, Infancy, Childhood

**Published:** 1887-09

**Authors:** 


MATERNITY, INFANCY, CHILDHOOD. Hygiene of Pregnancy; Nursing and Weaning of Infants; The Care of Children in Health and Disease. By John M. Keating, M. D. Philadelphia: J. B. Lippincott Co. 1887. Pp. 221. Price $1.00.

These manuals are not intended to be exhaustive treatises, but simply "Practical Lessons in Nursing." They are admirable for lay readers, presenting in convenient form many points of diet and nursing that physicians find can be with difficulty if at all impressed sufficiently on the patient and his friends.

Bruen's " Outlines for the Management of the Diet " is particularly clear and concise. Any intelligent person, guided by its teachings, can render very efficient aid to the doctor by giving the sick better nursing and careful, suitable feeding.

Keating's "Maternity, Infancy and Childhood" cannot be too highly recommended. It should be in the hands of every mother and every young married woman in the land. In this age of hasty, ill-assorted marriages, when prudish parents raise their daughters in ignorance and curse their grandchildren with disease by neglecting to give their own offspring proper advice and care, there is great need of such sensible instruction. Especially should the chapter on puberty be read by every mother, for here is given advice that, if heeded, would vastly diminish the female ills of the next generation. The intelligent physician will find this book to aid him greatly by increasing the hygienic care of his patients, thus providing that "ounce of prevention" which is better than a pound of cure.

